# Preparation of PCI Balloons: What Is the Best Method to Avoid Air in the Balloon? A Comparison of Different Methods of Connecting PCI Balloons and the Inflation Syringe while Removing Air from the Balloon

**DOI:** 10.3390/jcm9010172

**Published:** 2020-01-08

**Authors:** Laura Kreuser, Karl-Ludwig Laugwitz, Klaus Tiemann, Thorsten Lewalter, Clemens Jilek

**Affiliations:** 1 Internistisches Klinikum München Süd, Peter Osypka Herzzentrum, 81379 München, Germany; laura@kreuserteam.de (L.K.); klaus.tiemann@ikms.de (K.T.); Thorsten.lewalter@ikms.de (T.L.); 2 Klinik und Poliklinik Innere Medizin I (Kardiologie, Angiologie, Pneumologie), Klinikum rechts der Isar, Technische Universität München, 81379 München, Germany; laugwitz@mytum.de

**Keywords:** percutaneous coronary intervention (PCI), balloon inflation, balloon preparation, air remocal, acute coronary syndrome (ACS)

## Abstract

As the techniques to connect percutaneous coronary intervention (PCI) balloons and the inflation syringe vary in the instructions for use and in practice, we measured the amount of air in PCI balloons after testing three connection methods to an inflation syringe. Following the preparation using one of the three methods, 114 balloons and stent balloons were tested four times. Method 1 connected the syringe and the balloon catheter directly after purging and filling the lumen, while method 3 omitted the purging and filling process. With method 2, the catheter lumen was purged, filled and fully vented via a three-way valve. The primary endpoint answered whether air remained in the balloon, and if so, the secondary endpoint indicated the total volume of remaining air. The connection with a three-way valve achieved significantly less air in the inflated balloon as compared with either direct connection approach (27% *vs*. 44% and 51%; *p* = 0.015). For the direct connection, no significant difference between purging and filling the lumen prior to making the connection or not existed. According to these findings, the best method to connect a PCI balloon to the inflation syringe while removing air involves using a three-way valve.

## 1. Introduction

Percutaneous coronary intervention (PCI) is one of the existing standard techniques to treat stable coronary artery disease, non-ST-segment elevation acute coronary syndrome and ST-segment elevation myocardial infarction, according to the 2018 guidelines on myocardial revascularisation of the European Society of Cardiology and the European Association for Cardiothoracic Surgery [1].

During a PCI procedure, before the balloons or stents can be used, it is important to remove all air from the balloons. For this process, different manufacturers of PCI balloons and stents have provided a range of instructions [2,3,4,5]. In practice, in German heart catheter laboratories, differenttechniques are used. An informal survey among five heart catheter laboratories of primary, secondary and tertiary centres and different volumes showed that most of them do not follow the instructions of use. So, the question is also which consequences does this have regarding to air in the balloon. Even if in current age with the used balloons and contrast materials it seems less important to remove all the air from the balloon, it is still important to find the best method to avoid air in PCI balloons. Two points are important for success during the preparation process: first, the optimal method should leave no remaining air in the balloon; and, second, it should take as little time as possible to connect the balloon.

In this study, we tested three methods of connecting PCI balloons to the inflation syringe. The primary endpoint was whether air remained in the inflated balloon (“yes” or “no”). If a positive response was achieved, then the secondary endpoint of what was the total volume of remaining air (“high”, “medium” or “low”) was considered.

## 2. Material and Methods

We tested 114 new PCI or stent balloons of different lengths and diameters from four different producers. The lengths varied from 8.0 to 35.0 mm and the diameter from 1.25 to 5.0 mm. Table S1 shows the producers and models of the used balloons. We analysed the balloons and stents together and did not differentiate between them. In the following, the term “balloon” is used for both. To compare the different sizes of the balloons, four groups were established: group 1 contained short balloons with small diameters; group 2 contained long balloons with small diameters; group 3 contained short balloons with large diameters; and group 4 contained long balloons with large diameters. Table 1 shows the length and diameters of those groups.

We used the BasixTouchTM inflation syringe (Merit Medical Systems, South Jordan, UT, USA) for the first 41 balloons and the BasixCompakTM inflation syringe (Merit Medical Systems) for the remaining 73 balloons. The change in the model did not have an impact on the inflation process. Before each trial, the syringe was filled with 20 mL of the inflation solution.

We inflated the balloons with a mixture of iodine-based contrast media (ACCUPAQUE^TM^ 350 Iohexol; GE Healthcare, Chicago, IL, USA), which is used for coronary angiography, and 0.9% sodium chloride solution (1:2). The X-ray images were taken with the Allura Centron X-ray machine (Philips, Amsterdam, the Netherlands) in the heart catheter laboratory of the Internal Hospital Munich South (Internistisches Klinikum München Süd) in Munich, Germany.

**Figure 1:**
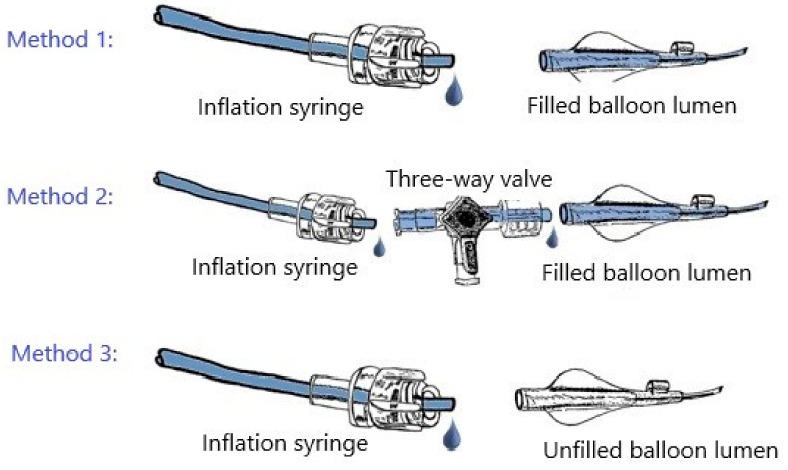
Methods of connecting the balloons.

We connected the balloons using one of three methods with the inflation syringe (Figure 1). These methods were created using information from the different manufacturers’ product manuals. We chose one set of instructions from each producer as exemplary [2,3,4,5].

Method 1 involved (1) purging and filling the balloon lumen with the inflation solution and connecting it to the inflation syringe and (2) aspirating three times for 10 s and then slowly releasing the negative pressure for 10 s. Method 2 involved (1) purging and filling the balloon lumen with the inflation solution; (2) connecting the inflation syringe and the balloon catheter via a three-way valve with the meniscus-to-meniscus method; (3) aspirating for 10 s and slowly releasing the negative pressure for 10 s; (4) removing air from the system through the three-way valve; and (5) again, aspirating and releasing the negative pressure for 10 s. Finally, method 3 involved (1) connecting the inflation syringe to the balloon catheter without purging and filling the balloon lumen and (2) aspirating three times for 10 s, then releasing the negative pressure for 10 s.

We attempted to use each method for the same number of times in each of the four groups of balloons. The assignment of the method to the single balloons was made by using random numbers in Microsoft Excel (Microsoft Corp., Redmond, WA, USA). The testing of the first 22 balloons (88 trials) was defined as the run-in period. This was to familiarise researchers with the methods and the use of the X-ray machine and to optimise the mixture of the inflation solution.

**Figure 2:**
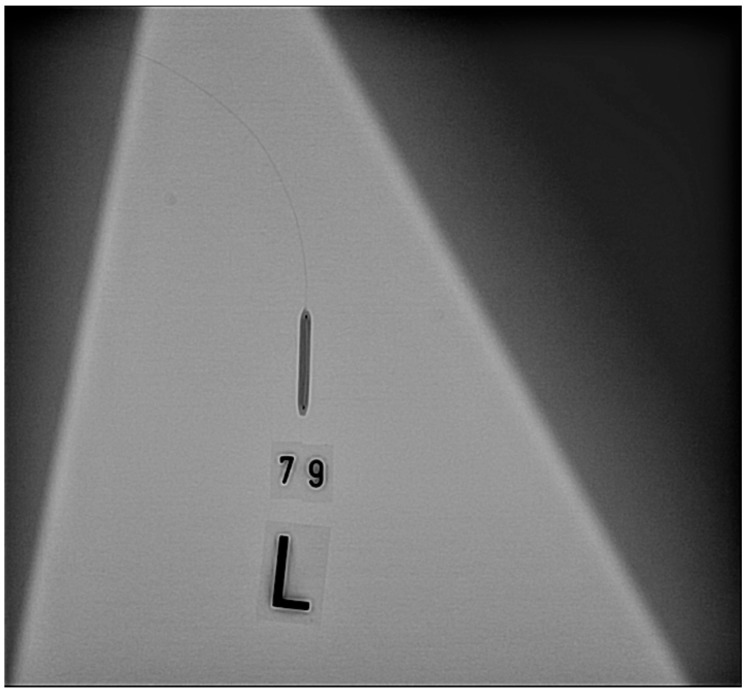
Balloon without air.

**Figure 3:**
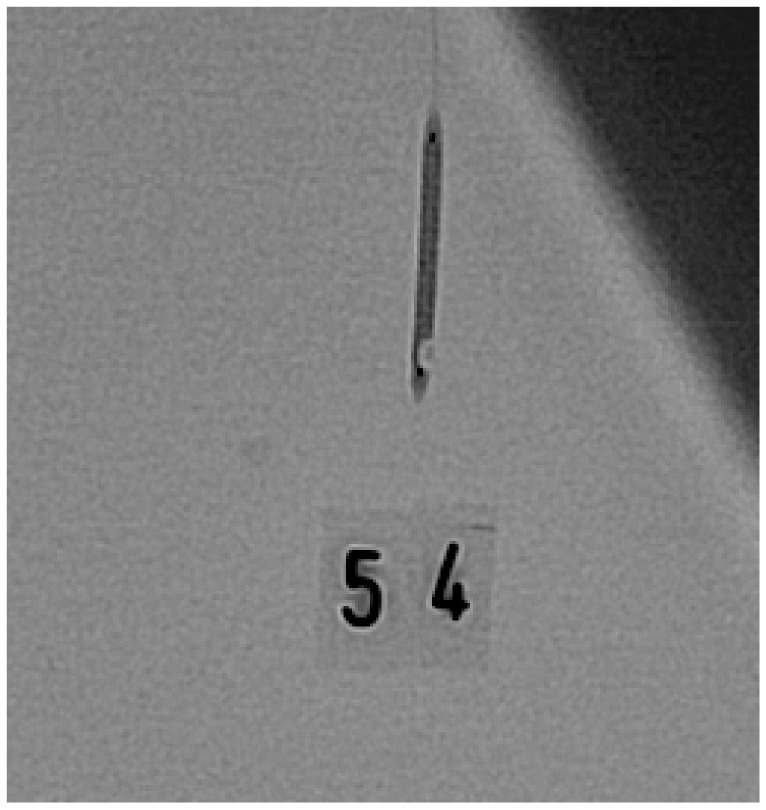
Balloon containing air.

**Figure 4:**
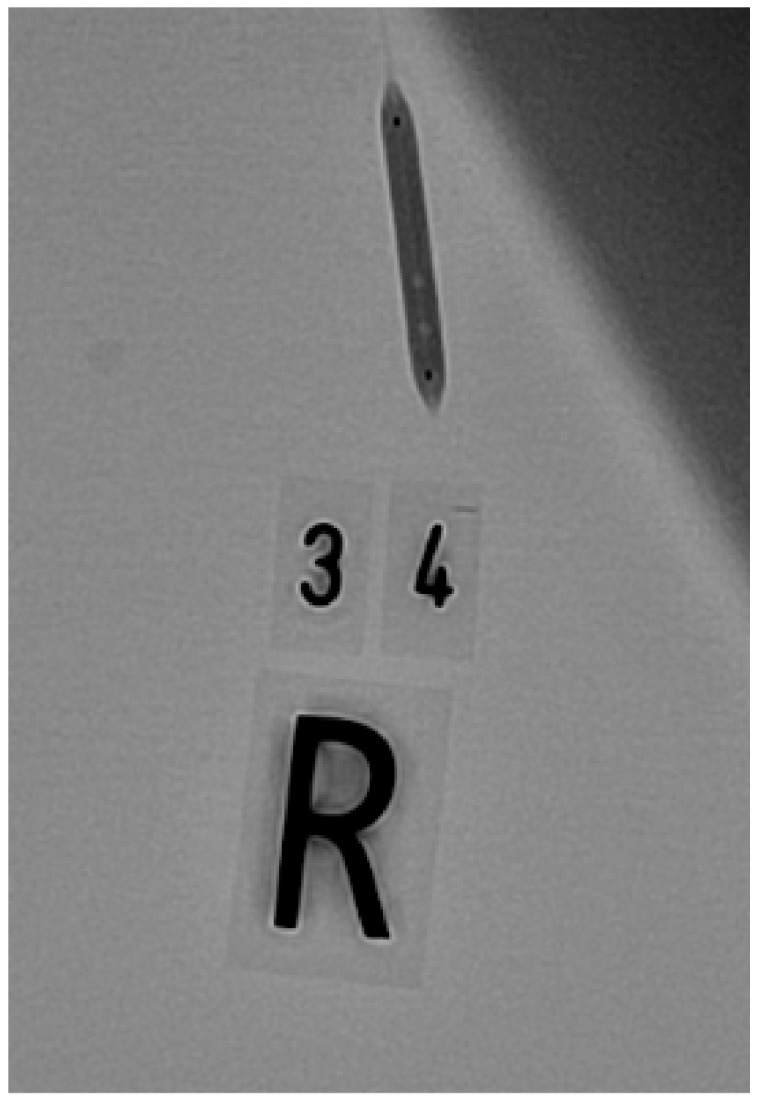
Balloon with two air bubbles.

Before connections were established using any of these methods, we filled the inflation syringe with 20 mL of the inflation solution and removed air from it, as indicated in the instructions for use [6]. Afterwards, the balloons were inflated to their nominal pressure (trials 1 and 2) or to 1 atm below their rated burst pressure (RBP) (trials 3 and 4). The balloons were inflated at a rate of 2 atm per second. We tested each balloon four times with the same method. Between inflations, we disconnected the inflation syringe and the balloon catheter and reconnected them. We changed the inflation syringe after 10 balloons (for the first 51 balloons) and after 20 balloons (for the last 63 balloons). The syringe change occurred due to a bottleneck in the supply chain. This bottleneck also necessitated a change in the syringe model after balloon 41. After every inflation, we took two X-ray images of the balloon (power: 25 pictures per second), including one in the posteroanterior 0/0° position and one in the left anterior oblique 40/0° position. Figure 2, Figure 3 and Figure 4 show one balloon without air and two balloons containing air, respectively.

We analysed the images using the programme IntelliSpace Cardiovascular version 1.2 [7]. As the primary endpoint, we looked for air in the balloon between the two metal markers (“yes” or “no”). For the secondary endpoint, we measured the length and width of the air bubbles and calculated the approximate volume. The volumes of the single bubbles were added to the total volume of air in the balloon. Based on these calculated total volumes, we defined three categories (“high”, “medium” and “low”) of total air volume. These categories can be seen in Table 2.

We statistically analysed our results using the SPSS Statistics version 25 software program (IBM Corp., Armonk, NY, USA). Generalised estimation equations with approved work correlation matrices were used for the analyses. The correlations were calculated using the Wald chi-squared test. The significance level was α = 0.05. Percent numbers containing decimals were rounded to whole numbers.

As sample size determination, we anticipated 20% of positive air in the balloon connected with a three-way valve and in the others with at least 40%, leading to a sample size per group of at least 81 tests with an alpha of 0.05 and a power of 80%.

## 3. Results

**Figure 5:**
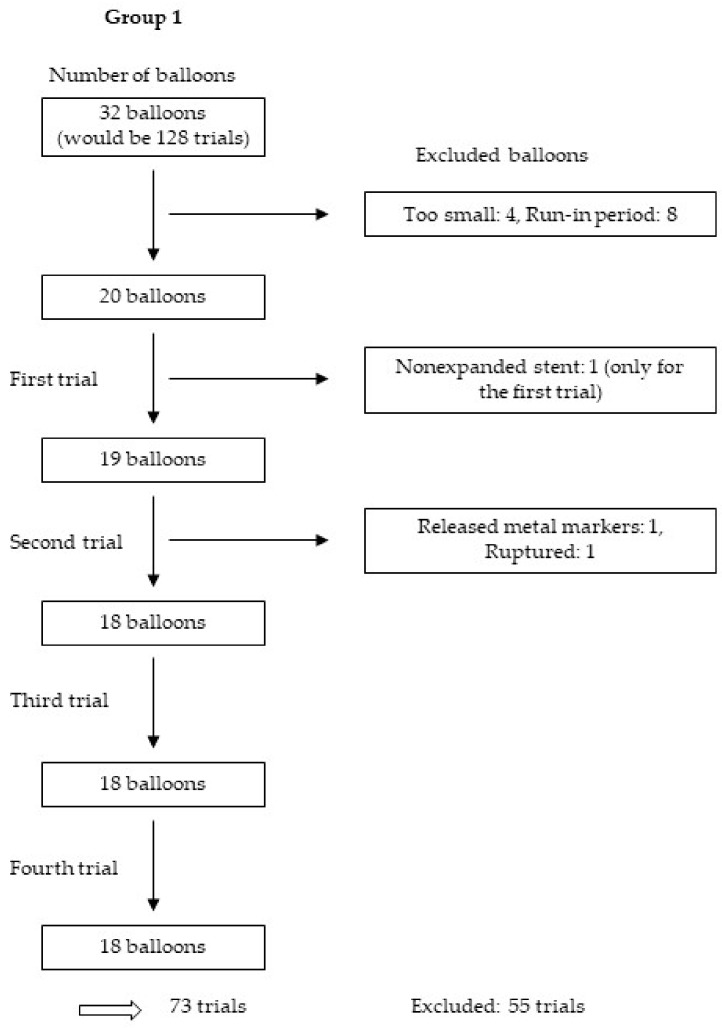
Excluded balloons group 1.

**Figure 6:**
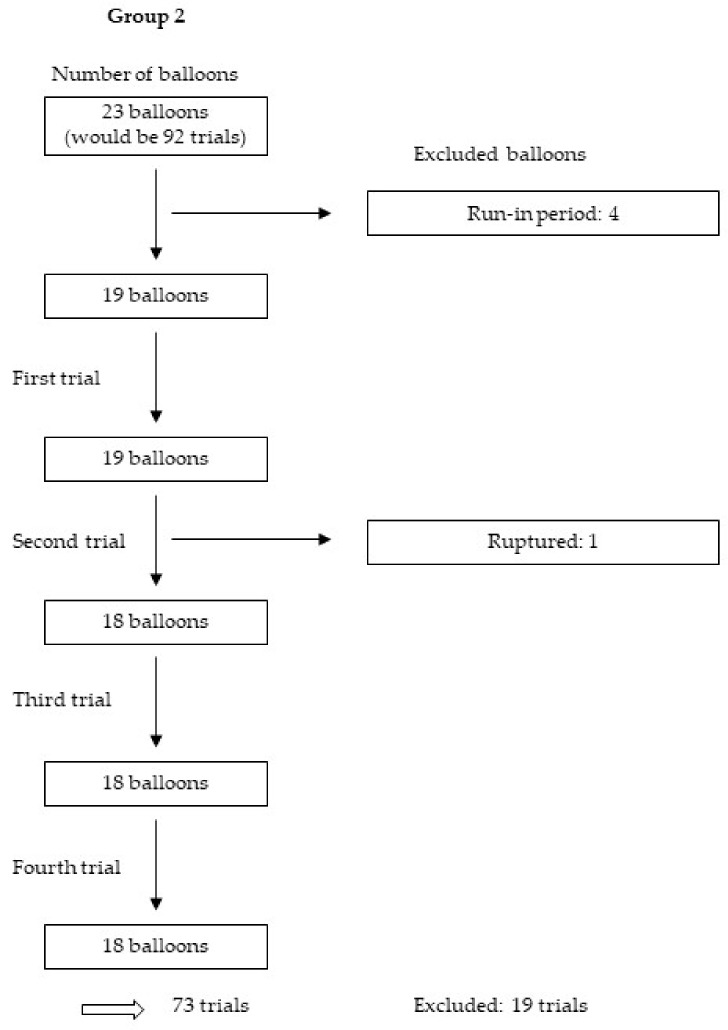
Excluded balloons group 2.

**Figure 7:**
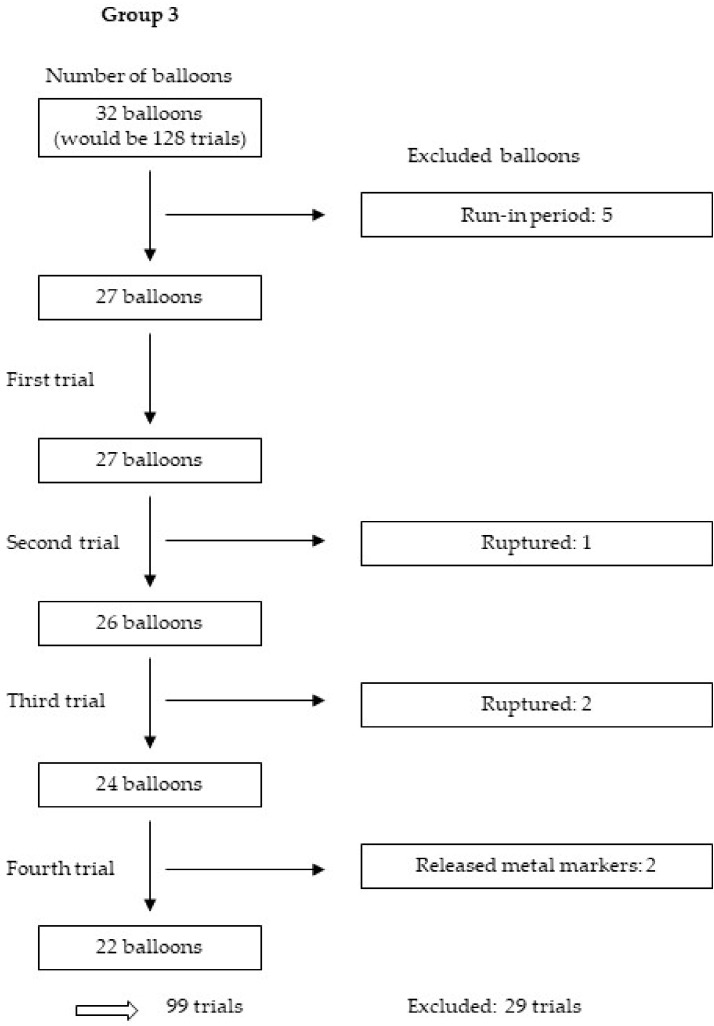
Excluded balloons group 3.

**Figure 8:**
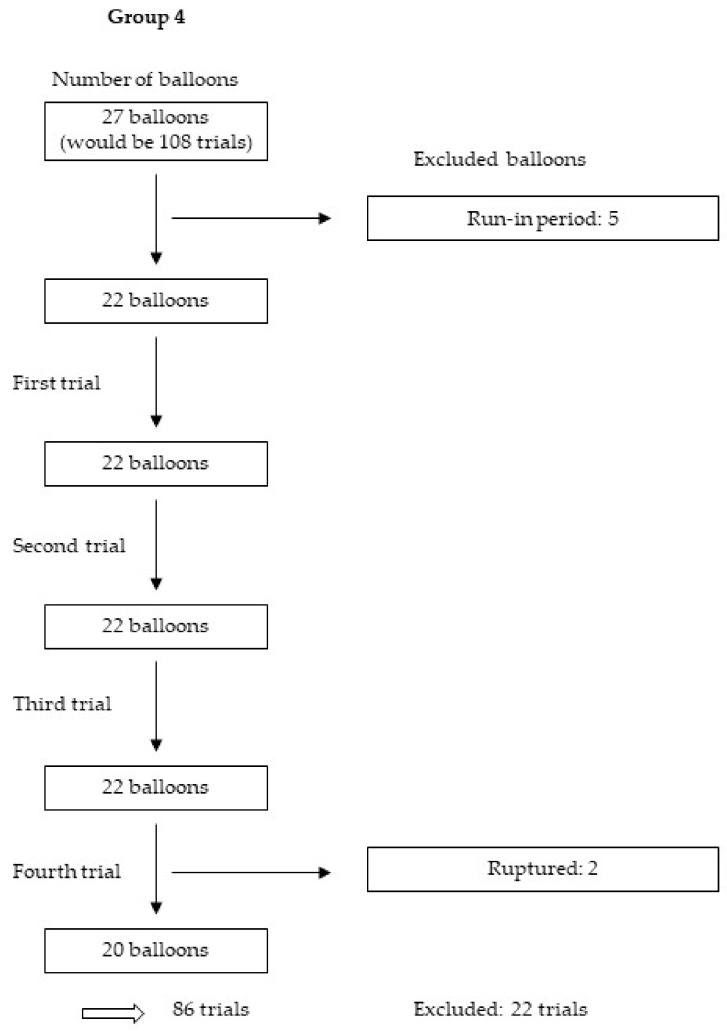
Excluded balloons group 4.

We tested 114 balloons four times each, yielding 456 trials. After the exclusion of 125 trials for various reasons, including those in the run-in period, as well as those with nonexpanded stents, balloons with released metal markers and balloons that were too small or ruptured, we analysed 331 trials from 30 balloons and 58 stents. Figure 5, Figure 6, Figure 7 and Figure 8 show the excluded balloons for each group. The excluded balloons were similar for each group except for group 1, which included the small and short balloons.

Air was found in 41% of all balloons. Connecting the balloon with a three-way valve (method 2) resulted in significantly less air than the approach of a direct connection including purging and filling the lumen (method 1) and a direct connection without purging and filling the lumen (method 3) (27% *vs*. 44% *vs*. 51%; *p* = 0.015; Table 3). No significant differences could be observed between methods 1 and 3 (Table 4).

A comparison of the four groups revealed that, in all four groups of balloons, the *p*-value for the differences between the three methods was significant only for group 4 (long balloons with large diameters). In the other groups, there were no significant differences between the methods (group 1: *p* = 0.468; group 2: *p* = 0.508; group 3: *p* = 0.361; group 4: *p* = 0.042; Table 5). Additionally, there was no significant interaction between groups and methods with respect to air in the balloon (*p* = 0.861). Furthermore, for the different pressures, there was only a significant difference between the three methods for the RBP-1 (nominal pressure: *p* = 0.222; RBP-1: *p* = 0.021; Table 6). No significant interaction was noted between the different pressure and methods with respect to air in the balloon (*p* = 0.389).

Additionally, no significant difference was found in the total volume between the three methods (*p* = 0.798; Table 7 and Table 8). The four groups of balloons also showed no significant difference in the total air volume in the three categories (group 1: *p* = 0.983; group 2: *p* = 0.333; group 3: *p* = 0.349; group 4: *p* = 0.638). There was also no significant interaction between the groups and methods with respect to the total volume of air (*p* = 0.322). Further, no significant difference was found for the different pressures (nominal pressure: *p* = 0.901; RBP-1: *p* = 0.785), and no significant interaction between the different pressures and methods with respect to the total volume (*p* = 0.947).

## 4. Discussion

According to our results, the best method to connect a balloon or stent in preparation for a PCI procedure and removing air from the balloon is by using a three-way valve. Although this method requires the most time, it leads to the least amount of air in the balloon. When a fast connection is used, it is not necessary to purge and fill the balloon; instead, it can be connected directly. The size of the balloon and the inflation pressure do not influence the presence of air in the balloon.

To our knowledge, this is the first study to compare different methods of connecting the inflation syringe specifically to a heart catheter balloon. A literature search in PubMed did not reveal any published results about different techniques of removing air from heart catheter balloons (Table S2). There is one study that compared six different preparation techniques to remove air from neurovascular angioplasty balloon catheters [8]. They also searched for a method to remove air from the balloons that is effective and simple to use [8]. The conclusion is that none of their methods lead to no air in the balloon, but the most simple and effective method would be to aspirate air for three times while using a 30 mL syringe [8]. This would be similar to our methods 1 and 3 (direct connections). In the described study, there was no comparison of filled or unfilled balloon lumens before connection. The study also used one method with a three-way-valve, aspiration for three times and disconnecting the syringe between the aspirations [8]. They showed that this methods leads to similar results as the other method but is less simple to proceed [8]. This method can be compared with our method 2 (using a three-way valve), but we did not disconnect the syringe between the aspirations and only vented the system via the three-way valve.

One of the complications that can be caused by using heart catheter balloons that still contain air is a coronary artery air embolism, which can lead to several symptoms ranging from mild angina to full cardiac arrest [9].

Most of the time, air embolism is caused by not properly flushed vascular catheters [10], but also if a balloon bursts, air can embolise in the blood system and create a perfusion deficit [9,10,11]. In our study, we measured a total air volume ranging from 0.03 to 18.0 mm^3^ in the included balloons. If a coronary artery air embolism occurs, the size of the air bubble can influence the severity of the cardiac depression [11,12,13]. In the published literature, there are very different volumes of air that cause severe clinical cardiac symptoms; in one study in particular, volumes from 2000 to 3000 mm^3^ led to severe life-threatening symptoms [13]. We did not approach this volume in our study. Another study with dogs showed a mortality rate of 28% in dogs after injection of 0.02 mL air/kg of body weight [14]. Other researchers demonstrated increasing cardiac depression in pigs by injecting air bubbles with approximate volumes of 0.002 mm^3^ (diameter: 75 µm), 0.014 mm^3^ (diameter: 150 µm) or 0.113 mm^3^ (diameter: 300 µm) [12]. As compared with this, we measured a very high total volume of air (up to 18.0 mm^3^), which would most likely cause a considerable cardiac depression. The volume of the air bubble that produces a blood flow obstruction in the coronary artery is also dependent on the size of the artery containing the air bubble, the mean arterial pressure, local shear stress where the air bubble is located, myocardial contraction and partial pressure of arterial oxygen and nitrogen partial pressures [15]. In addition to the injected air volume, the severity of the produced cardiac depression is determined by the baseline cardiac function and caused vascular responses like distal air-lock or the presence of vasospasm [10].

However, the animal data show how important it is for PCI procedures to use a method to connect the balloon without retaining air. The best way to treat coronary artery air embolism is to prevent it from happening entirely [9,10,11].

Another complication of PCI procedures is incomplete stent apposition (ISA), also called stent malapposition [16]. This term describes the presence of incomplete contact between the stent after its expansion and the intimal surface of the vessel wall [17]. Acute ISA can be caused by several factors, although technical aspects are the main cause of acute ISA [16]. Air in the balloon might constitute one of these technical aspects. The inflation solution is not compressible in contrast with air, which could eventually lead to an incomplete expansion of the stent struts where the air bubble is located. For this hypothesis, we conducted a literature search in PubMed, but could not find any studies on the relationship between air in the balloon and ISA (Table S3).

A limitation of this study is that a large percentage of balloons were found to contain air. In our research, we searched in detail for air in the balloons. We even noticed very small air bubbles. These may also appear in daily clinical practice, but no one searches explicitly for them and so they usually remain undetected. Another aspect that could have led to more air bubbles in the balloons was that the contrast media we used was at room temperature. Normally, in a PCI procedure, the contrast media should be heated to 37 °C. The temperature may influence the physical characteristics of the contrast media: the lower the temperature the higher the viscosity [18]. We used a modern low-viscosity contrast medium, nevertheless, the temperature alone could have led to increased volume of air in the balloons. The fact that we measured more air bubbles in the present research than are typically found in a clinical PCI procedure should not influence the main findings of this study because we tested every balloon and every balloon connection under the same conditions.

## 5. Conclusions

We recommend using a three-way valve to connect the balloon catheter and the inflation syringe during PCI procedures. With respect to the volume of air in the balloon, if the inflation syringe and the catheter are connected directly, the establishment of a connection without purging and filling the balloon lumen is as effective as purging and filling the balloon lumen before the connection is made. However, it remains unclear whether this difference is clinically meaningful, even if the difference in air volume between the methods is statistically significant.

## Figures and Tables

**Table 1:** Groups of balloons and stents.

		Diameter	Length
**Balloons**	Group 1	≤2.5 mm	≤18.0 mm
	Group 2	≤2.5 mm	≥20.0 mm
	Group 3	≥3.0 mm	≤18.0 mm
	Group 4	≥3.0 mm	≥20.0 mm

**Table 2:** Total volume categories.

		Volume in mm^3^
**Total volume category**	High	≥1.35
	Medium	≥0.51, <1.35
	Low	<0.51

**Table 3:** Air in the balloons depending on the method.

		Air	
		Yes	No
**Method**	1 (direct connection with filling the lumen)	44%	56%
	2 (three-way valve)	27%	73%
	3 (direct connection without filling the lumen)	51%	49%
**Total**		41%	59%

**Table 4:** Pairwise comparison of the methods.

				95% Confidence Interval	
(I) Method	(J) Method	Mean Difference (I-J)	Significance	Lower Value	Upper Value
1 (direct connection with filling the lumen)	2	−0.17	0.028	−0.33	−0.02
	3	0.08	0.320	−0.08	0.23
2 (three-way valve)	1	0.17	0.028	0.02	0.33
	3	0.25	0.002	0.09	0.41
3 (direct connection without filling the lumen)	1	−0.08	0.320	−0.23	0.08
	2	−0.25	0.002	−0.41	−0.09

**Table 5:** Air in the balloons for the different groups.

		Air	
Group	Method	Yes	No
1 (short, small diameter)	1 (direct connection with filling the lumen)	46%	54%
	2 (three-way valve)	29%	71%
	3 (direct connection without filling the lumen)	47%	53%
	Total	40%	60%
2 (long, small diameter)	1 (direct connection with filling the lumen)	38%	62%
	2 (three-way valve)	19%	81%
	3 (direct connection without filling the lumen)	39%	61%
	Total	33%	67%
3 (short, large diameter)	1 (direct connection with filling the lumen)	35%	65%
	2 (three-way valve)	34%	66%
	3 (direct connection without filling the lumen)	51%	49%
	Total	41%	59%
4 (long, large diameter)	1 (direct connection with filling the lumen)	56%	44%
	2 (three-way valve)	22%	78%
	3 (direct connection without filling the lumen)	63%	37%
	Total	48%	52%

**Table 6:** Air in the balloons for the different pressures.

		Air	
Pressure	Method	Yes	No
Nominal pressure	1 (direct connection with filling the lumen)	38%	62%
	2 (three-way valve)	28%	72%
	3 (direct connection without filling the lumen)	44%	56%
	Total	37%	63%
Rated burst pressure-1	1 (direct connection with filling the lumen)	50%	50%
	2 (three-way valve)	25%	75%
	3 (direct connection without filling the lumen)	58%	42%
	Total	45%	55%

**Table 7:** Total volume of air depending on the method.

		Volume Category		
		High	Medium	Low
**Method**	1 (direct connection with filling the lumen)	27%	38%	35%
	2 (three-way valve)	32%	32%	36%
	3 (direct connection without filling the lumen)	39%	28%	33%
**Total**		34%	32%	34%

**Table 8:** Mean total volume depending on the method.

		Mean Total Volume (mm^3^)
**Method**	1 (direct connection with filling the lumen)	1.12
	2 (three-way valve)	1.25
	3 (direct connection without filling the lumen)	2.25
**Total**		1.64

## Supplementary Materials

The following are available online at https://www.mdpi.com/2077-0383/9/1/172/s1, Table S1: Balloons and stents—producers and models, Table S2: Literature search results for terms involving or relating to “connection”, Table S3: Literature search results for terms involving or relating to “stent malapposition”.
