# Supplementary material for: Preparation of PCI Balloons: What Is the Best Method to Avoid Air in the Balloon? A Comparison of Different Methods of Connecting PCI Balloons and the Inflation Syringe while Removing Air from the Balloon
Source: J Clin Med. 2020 Jan 8;9(1):172. doi: 10.3390/jcm9010172 (PMC7020097; doi:10.3390/jcm9010172)
Supplement: Supplementary file 1 [file jcm-09-00172-s001.pdf]

Supplementary Materials (Preparation of PCI Balloons)

Table S1. Balloons and stents – producers and models

	Producer	Model	Number
**Balloons**	B. Braun	SeQuent NEO	13
	Medtronic	NC Sprinter	1
		Sprinter Legend	5
		NC Solarice	4
	OrbusNeich	Sapphire	3
		Sapphire II	9
	Biotronik	Pantera	3
		Pantera Pro	8
		Pantera LEO	1
**Stents**	B.Braun	Coroflex Blue	1
		Coroflex Blue Neo	11
		Coroflex Blue Ultra	6
	Medtronic	Integrity	3
		Resolute Integrity	6
		Resolute Onyx	4
	OrbusNeich	Azule	3
		COMBO	18
	Biotronik	PRO-Kinetic Energy	9
		Orsiro	6
**Total number**			114

Table S2. Literature search results for terms involving or relating to 'connection'

pci air	pci air bubble	pci air removal
	pci 'air bubble'	pci 'air removal'
pci balloon air	pci balloon preparation	pci balloon purging
	pci 'balloon preparation'	pci 'balloon purging'
heart catheter air	heart catheter air bubble	heart catheter air removal
'heart catheter' air	heart catheter 'air bubble'	heart catheter 'air removal'
	'heart catheter' air bubble	'heart catheter' air removal
	'heart catheter' 'air bubble'	'heart catheter' 'air removal'
heart catheter balloon air	heart catheter balloon preparation	heart catheter balloon purging

‘heart catheter’ balloon air	heart catheter ‘balloon preparation’	heart catheter ‘balloon purging’
‘heart catheter balloon’ air	‘heart catheter’ balloon preparation	‘heart catheter’ balloon purging
	‘heart catheter’ ‘balloon preparation’	‘heart catheter’ ‘balloon purging’
	‘heart catheter balloon’ preparation	‘heart catheter’ balloon purging

Table S3. Literature search results for terms involving or relating to ‘stent malapposition’

stent malapposition mechanisms	incomplete stent apposition mechanisms
‘stent malapposition‘ mechanisms	‘incomplete stent apposition‘ mechanisms
stent malapposition causes	incomplete stent apposition causes
‘stent malapposition‘ causes	‘incomplete stent apposition‘ causes
stent malapposition air	incomplete stent apposition air
‘stent malapposition‘ air	‘incomplete stent apposition‘ air
stent mal apposition mechanisms	isa air
‘stent mal apposition‘ mechanisms	isa pci air
stent mal apposition causes	isa mechanisms
‘stent mal apposition‘ causes	isa pci mechanisms
stent mal apposition air	isa pci causes
‘stent mal apposition‘ air	
